# Terminal Genome Sequences of the Soft Tick Bunyavirus

**DOI:** 10.1128/MRA.00126-20

**Published:** 2020-04-30

**Authors:** Satoko Sugimoto, Yuto Suda, Tomoki Yoshikawa, Takeshi Kurosu, Tetsuya Mizutani, Masayuki Saijo, Masayuki Shimojima

**Affiliations:** a Cooperative Division of Veterinary Sciences, Graduate School of Agriculture, Tokyo University of Agriculture and Technology, Tokyo, Japan; b Department of Veterinary Microbiology, Graduate School of Agricultural and Life Sciences, University of Tokyo, Tokyo, Japan; c Department of Virology I, National Institute of Infectious Diseases, Tokyo, Japan; Portland State University

## Abstract

The complete genome sequence of the soft tick bunyavirus (STBV) was obtained using the Sanger sequencing technique. Comparison with other viral sequences revealed that STBV has unique sequences in the terminal regions that are highly conserved among the genus *Orthonairovirus*.

## ANNOUNCEMENT

The soft tick bunyavirus (STBV) was originally isolated by Oba et al. (1) from a pool of soft ticks, Argas vespertilionis, collected in feces beneath bat colonies located in a human habitat in Japan (2). A next-generation sequencing analysis revealed that the STBV genome consists of three negative-strand RNA segments (1). The virus is most closely related to Keterah virus isolated in Malaysia (3), which is a member of the species Keterah orthonairovirus of the genus *Orthonairovirus* of the family *Nairoviridae.* The sequence of STBV deposited in GenBank (accession no. LC027465 to LC027467) lacked the 3′ and 5′ terminal sequences in each segment (24 and 20 nucleotides in segment L, 24 and 17 nucleotides in segment M, and 228 and 23 nucleotides in segment S at the 3′ and 5′ termini, respectively). Previously, it was recognized that each segment of *Orthonairovirus* genomes has nine complementary terminal consensus sequences, 3′ terminus AGAGUUUCU and 5′ terminus AGAAACUCU (4). However, Kuhn et al. (3) reported that several recently identified *Orthonairovirus* members have consensus regions that differ by one or two nucleotides. Here, we determined the complete genome sequence, including the terminal regions of STBV, and compared the terminal sequences with those of other *Orthonairovirus* members.

STBV was passaged three times in Vero cells and once in BME/CTVM2 cells (5). The viral RNA was extracted from the culture supernatant using a High Pure viral RNA kit (Roche Applied Science, Mannheim, Germany). To obtain DNA corresponding to the viral terminal regions, rapid amplification of cDNA ends (RACE) (6) was performed. Virus-specific primers used in RACE were as follows: CCCCAGTAATCATCTCTC for the L segment, TCTCTGTGTCCACTGTTC for the M segment, and GAAGCAGAGAGAGTTGCT for the S segment at the 3′ termini and GGACTAATCTAATCTGCGGC for the L segment, GGAACACCGCAGTACTATCT for the M segment, and GGCTTCTACCTGCACTAACA for the S segment at the 5′ termini. The amplified products were directly used for sequencing without cloning using Sanger’s method (7) with an Applied Biosystems 3500xL genetic analyzer (Thermo Fisher Scientific) according to the manufacturer’s protocol. The complete sequences of the STBV L, M, and S segments were deposited in GenBank under accession no. LC495731, LC495732, and LC495733, respectively.

Although a few differences were detected in the L segment (3 nucleotides) and S segment (13 nucleotides), the determined sequences were almost the same as those in the previously reported sequences, except for the termini (1). The terminal 9-nucleotide sequences of STBV were not the same as those of the consensus sequences of the genus *Orthonairovirus* (4), and noncomplementary nucleotide pairings at the fifth-most-terminal nucleotide were observed in the L and M segments (Table 1). We observed the same terminal sequences in the Issyk-Kul virus (ISKV) propagated in SW-13 cells by applying the strategy for sequencing described above (GenBank accession no. LC495734 to LC495736); terminal nucleotides of ISKV were identical to those reported by Atkinson et al. using the virus propagated in a suckling mouse (8). Thus, the terminal sequence appears to well reflect the reported phylogenetic tree (3). Similar terminal sequences with one or two mismatched pairings were observed for STBV-related viruses, e.g., Qalyub virus and Dera Ghazi Khan virus (Table 1).

**TABLE 1:** The nine terminal nucleotide sequences of viral genomes in the genus *Orthonairovirus*^*a*^

Species	Virus	Sequence for^*b*^:						Reference or accession no.^*d*^
		L segment		M segment		S segment		
		3′	5′	3′	5′	3′	5′	
	Consensus sequence	AGAGUUUCU	AGAAACUCU	AGAGUUUCU	AGAAACUCU	AGAGUUUCU	AGAAACUCU	4
Keterah	Keterah virus	NA	NA	AGAGUUUCU	NA	NA	NA	KR537447, KR537448, KR537449
Keterah	Soft tick bunyavirus^*c*^	AGAGUUUCU	AGAA**U**CUCU	AGAGUUUCU	AGAA**U**CUCU	AGAG**A**UUCU	AGAA**U**CUCU	LC495731, LC495732, LC495733
Keterah	Issyk-Kul virus^*c*^	AGAGUUUCU	AGAA**U**CUCU	AGAGUUUCU	AGAA**U**CUCU	AGAG**A**UUCU	AGAA**U**CUCU	KR709221, KR709220, KR709219
Qalyub	Qalyub virus	AGAG**A**UUCU	AGAAACUCU	AGAG**A**UUCU	NA	AGAG**A**UUCU	AGAA**U**CUCU	KU925476, KU925477, KU925478
Dera Ghazi Khan	Dera Ghazi Khan virus	AGAGUUUCU	AGAAACUCU	AGAGUUUC**A**	AGAAACUCU	AGAGUUUC**A**	AGAAACUCU	KU925452, KU925453, KU925454
Dera Ghazi Khan	Abu Hammad virus	AGAGUUUC**A**	**U**GAAACUCU	AGAGUUUC**A**	**U**GAAACUCU	AGAG**A**UUC**A**	**U**GAAACUCU	KU925434, KU925435, KU925436
Dera Ghazi Khan	Abu Mina virus	AGAGUUUC**A**	**U**GAAACUCU	AGAGUUUC**A**	**U**GAAACUCU	AGAG**A**UUC**A**	**U**GAAACUCU	KU925437, KU925438, KU925439
Dera Ghazi Khan	Tunis virus^*c*^	AGAGUUUC**A**	**U**GAAACUCU	AGAGUUUC**A**	**U**GAAACUCU	AGAG**A**UUC**A**	**U**GAAACUCU	KU925497, KU925498, KU925499
Dera Ghazi Khan	Sapphire II virus^*c*^	AGAGUUUC**A**	**U**GAAACUCU	AGAGUUUC**A**	**U**GAAACUCU	AGAGUUUC**A**	**U**GAAACUCU	KU925485, KU925486, KU925487

a Shown are members of the genus *Orthonairovirus* whose terminal nine nucleotides in their genomes are different from the consensus sequences (4).

b NA, not available; bold, different bases from the consensus; underline, noncomplementary nucleotide pairs.

c Proposed classification by Kuhn et al. (3).

d Accession numbers are given for the L, M, and S segments, respectively.

In summary, a full-genome sequence, including the termini of STBV was determined using Sanger’s method. The STBV has the same terminal sequences as ISKV. Determination of terminal sequences of viral genomes might accelerate the classification of *Orthonairovirus* members.

### Data availability.

The sequences determined in the present study have been deposited under the accession no. LC495731, LC495732, and LC495733 (STBV) and LC495734, LC495735, and LC495736 (ISKV).
